# Relationship in Japan between maternal grandmothers’ perinatal support and their self-esteem

**DOI:** 10.1111/nhs.12079

**Published:** 2013-07-01

**Authors:** Atsuko Iseki, Kazutomo Ohashi

**Affiliations:** 1 School of Nursing, Faculty of Medicine, Mie UniversityMie; 2 Department of Children's and Women's Health, Area of Nursing Science, Division of Health Sciences, Osaka University Graduate School of MedicineOsaka, Japan

**Keywords:** cross-cultural healthcare, depression, grandmother, Japan, perinatal support, self-esteem

## Abstract

This study investigated the influence on their mental well-being of the perinatal support given by Japanese grandmothers.

The Rosenberg self-esteem and the Center for Epidemiologic Studies Depression (CES-D) scales were used to assess grandmothers’ mental well-being before and after their daughters’ childbirth. Of 198 grandmothers, 176 (88.9%) supported their daughters and three patterns of perinatal support were observed: grandmothers’ support at the grandparents’ house before childbirth (*n* = 95) (Satogaeri bunben; Japanese traditional perinatal support), grandmothers’ support at the grandparents’ house after childbirth (*n* = 53); and grandmothers’ support at the daughters’ house (*n* = 28).

Those who supported their daughters at the grandparents’ house before childbirth – especially the middle-aged (less than 60 years old) – showed significantly lower scores of self-esteem. Scores of CES-D did not significantly change before and after childbirth in either subgroup of grandmothers. It was concluded that grandmothers play an important role in supporting their daughters, and Satogaeri bunben is a typical event in modern Japan. However, Satogaeri bunben is a burden for middle-aged grandmothers, and we need to support them.

## Introduction

A grandmother plays an important role during the period around her daughter's childbirth. Emotional and psychological supports regardless of geographic proximity are the most frequent forms of perinatal support (Martell, 1990). Although grandmothers' support affects their daughters and grandchildren, the effects of such supports on the grandmothers themselves remain unclear.

### Literature review

A grandmother may provide breastfeeding advice to her daughter (Grassley & Eschiti, 2008) and this advice has a potential influence on decision-making about the mother's child-rearing method (Reid *et al*., 2009). Several studies have examined the effects of grandmothers' support of their daughters on the grandchildren. Grandparents' involvement in caregiving affects maternal and child health (Arnold *et al*., 2010). Significant support from grandparents has been associated with positive infant attachment (Spieker & Bensley, 1994). Child behavioral problems have been shown to be no greater in either mother–grandmother or mother–relative families compared with intact nuclear families (Simons *et al.*, 2006). There are also some studies of the effects of grandmothers' support on the grandmothers themselves. If the mother is an adolescent (Sadler & Clemmens, 2004) or resides in prison (Baker *et al.*, 2010), the grandmother may become the main care-giver instead of the parents. When grandmothers raise their grandchildren, this can create high stress levels (Dowdell, 2004).

Support systems for post-partum women involves cultural factors and traditions that are specific to each country (Eberhard-Gran *et al.*, 2010). China has a unique support system named “Doing the Month” in which women after childbirth should obey some prohibitive rules about diet and behavioral patterns including bodily cleaning while receiving the grandmother's care (Eberhard-Gran *et al.*, 2010; Holroyd *et al.*, 2011). Several adverse effects on maternal health have been found to result from this practice (Liu *et al.*, 2012). In Korea, it has been traditional for the post-partum mother to receive the grandmother's care. Currently, most mothers in Korea receive such services from a private institution named “Sanfujori center” (Kim, 2003). One of the Japanese customs of perinatal care is “*Satogaeri bunben*” in Japanese (Yoshida *et al.*, 2001; Eberhard-Gran *et al.*, 2010; Kobayashi, 2010). A mother returns to her parent's house in late pregnancy, and receives support from her family members during the perinatal and postnatal periods. This Japanese custom is conventionally thought to have a good influence on the mother's mental health. The significance of *Satogaeri bunben* for the mother in the puerperal period is considered to promote acquisition and learning of parenting techniques and a reduction of anxiety about child care (Kobayashi, 2010). While the grandmother plays an important role in *Satogaeri bunben*, there have been few studies on the effects or burden on the grandmother.

Self-esteem is an index of psychological well-being and is a self-concept related to self-efficacy (Hughes *et al.*, 2004), quality of life (Kim *et al.*, 2009), and tendency to experience depression (Napholz & Mo, 2010). Low self-esteem is associated with low socioeconomic conditions, including low income, low education level, and delinquency (Reitzes & Mutran, 2002; 2004, ). Recently, some life events, for example, domestic violence (Loke *et al.*, 2012) or hysterectomy (Pinar *et al.*, 2012), were reported to be associated with low levels of self-esteem. In general, supporting others is associated positively with the level of self-esteem of the supporters (Midlarsky, 1991; Krause & Shaw, 2000) and reduced levels of depression of the supporters (Krause *et al.*, 1992). If a grandmother satisfies her role in supporting her daughter, it may be considered that she might have a high level of self-esteem and reduced levels of depression. Moreover, the level of self-esteem shows a positive correlation between a grandmother and her daughter (Sadler & Clemmens, 2004), indicating that grandmothers possibly influence high levels of self-esteem in mothers.

### Study aim

The aim of this study was to determine the correlation between the perinatal support provided by grandmothers and their mental well-being. We examined the self-esteem and tendency to experience depression in grandmothers who support their daughters before and after childbirth.

## Methods

### Study design

This study was conducted in three private obstetric clinics in Mie prefecture, central Japan, from May 2009 to October 2010. These clinics see 600 to 800 births per year. Participants included the mothers of pregnant women who visited the clinics for antenatal care and were named as grandmothers in the study. The study comprised two sets of investigations using questionnaires. The first study was conducted before the daughter's childbirth and the second following the daughter's childbirth.

### Participants and data collection

Nursing staff or obstetricians asked the pregnant women to participate in the study while they were waiting for a prenatal examination after 28 weeks of gestation. After obtaining written informed consent from the pregnant women, an explanation of the study, a written request for participation, a written informed consent form for the grandmother and questionnaires for the two investigations were handed to each pregnant woman. The pregnant women handed these documents to their mothers directly or sent them by mail. The completed first questionnaires and written informed consent form were returned to an investigator (Iseki) by the grandmothers by mail or were brought to the clinics by the pregnant women on behalf of the grandmothers. Questionnaires that were completed by 12 weeks after childbirth were sent by the grandmothers directly by mail.

### Instruments

#### Questionnaire

The questionnaire consisted of social background factors of grandmothers, pre- and perinatal information, self-esteem scales, and depression scales. The social background factors of grandmothers investigated before childbirth (the first examination) were age, marital status, education, employment status, hobbies and volunteer activities, perceived financial burden, perceived health status, and daily care for family members. The grandmother's daughter's information including parity and gestational week were also obtained. After childbirth (the second examination), the type of delivery and type of grandmothers' support were examined. The place and time of grandmothers' support was divided into three types: at the grandparents' house before childbirth, at the grandparents' house after childbirth, and at the daughter's house. The grandmother's support at the daughter's house was just postnatal support and did not include prenatal support. Cases with no grandmother support were also analyzed.

#### Self-esteem

Self-esteem was measured by the Rosenberg self-esteem scale (Rosenberg, 1985). This scale consists of five items of positive attitude and five items of negative attitude. The participants can select responses of “strongly agree”, “agree”, “disagree”, and “strongly disagree”. Scores for positive attitude ranged from 4 to 1, while negative attitude scored 1 to 4. The sum of scores ranged from 10 to 40 and higher scores indicated higher levels of self-esteem.

The Rosenberg self-esteem Japanese version (RSES-J) (Mimura & Griffiths, 2007) was used in the study. Based on results from the European Health Research Group on Health Outcome Recommendation (Meadows *et al.*, 1996) and International Test Commission Guidelines (Van de Vijuver & Hambleton, 1996), the scale was translated into Japanese by four married couples, each comprising a Japanese and a Briton, with a forward–backward translation method. After translation, the variability and reliability were confirmed using 1320 university students. The resulting RSES-J was a two-factor structure similar to the original scale. The Cronbach's alpha value of the first factor was 0.76 and that of the second factor was 0.67. In our preliminary study (Iseki & Ohashi, 2011), the RSES-J was used to estimate the self-esteem of 207 women aged from 40 to 70 years in Mie prefecture. The RSES-J also showed the two-factor structure and the Cronbach's alpha values of factors 1 and 2 were 0.71 and 0.69, respectively.

#### Depressive condition

The Center for Epidemiologic Studies Depression (CES-D) Scale was developed to assess the depressive condition and consists of 20 items concerning the perception of mental and physical depressive symptoms (Radloff, 1977). Options for the frequency of symptoms during the past week were “none”, “one or two days”, “three or four days,” or “four days or more”, rating 0, 1, 2 and 3, respectively. The range of total sum scores was from 0 to 60 and a high score indicated a severe depressive episode. The CES-D Japanese version was developed by Shima *et al*. (1985). The cut-off point to detect depression is 16 in the CES-D Japanese version.

### Data analyses

Data analysis was performed with SPSS for Windows Version 17. The Fisher's exact test was used to examine the relationship between characteristics of grandmothers and presence of grandmother's support. Scores of self-esteem and depression before and after childbirth were analyzed using the paired *t*-test. Scores of self-esteem and CES-D before childbirth were compared among support patterns using Tukey's multiple comparison tests. The significance level was set at *P* < 0.05.

### Ethical considerations

This study was approved by The Ethics Committee of Mie University Medical School in July 2008 (No.990). The anonymity of participants was preserved and no financial burden was placed on them.

## Results

Of 600 grandmothers, 237 (39.5%) sent both questionnaires and 216 (36.0%) fully responded to the CES-D and self-esteem scales. Of the 216, 18 were excluded from the study as participants answered the first questionnaire before 28 weeks of gestation or the second study 13 weeks or more after their daughters' childbirth. Finally, 198 (33.0%) responses were analyzed. All daughters experienced birth at term.

Characteristics of the grandmothers are shown in Table 1. The mean age of grandmothers was 57.0 ± 5.3. The number of grandmothers aged less than 60 years (middle-aged group) was 130 (65.7%) and the numbers aged 60 or more (older group) was 68 (34.3%). The percentages of grandmothers who supported their daughters during the perinatal period were not different between the two age groups. Marital status, education, employment status, hobby and volunteer activity, perceived financial burden, perceived health status, and daily care for family members were not associated with grandmother's support. Only parity of their daughters was related to support, with more primiparous women receiving support than multiparous women (*P* = 0.024).

**Table 1:** Characteristics of grandmothers

Characteristic	Support of grandmothers			*P*-value
	Total	Present (*n* = 176)	Absent (*n* = 22)	
	N	N (%)	N (%)	
Age				
< 60 years (middle aged)	130	114 (87.7)	16 (12.3)	0.635
≥ 60 years (elderly)	68	62 (91.2)	6 (8.8)	
Marital status				
Married	176	156 (88.6)	20 (11.4)	1.000
Divorced or widowed	22	20 (90.9)	2 (9.1)	
Education†				
High school or lower	150	133 (88.7)	17 (11.3)	1.000
College or higher	47	42 (89.4)	5 (10.6)	
Employment status				
Housekeeping	88	81 (92.0)	7 (8.0)	0.258
Employed	110	95 (86.4)	15 (13.6)	
Hobbies and volunteer activities				
Regular	87	79(90.8)	8 (9.2)	0.501
Irregular or none	111	97 (87.4)	14 (12.6)	
Perceived financial burden				
Absent	162	146 (90.1)	16 (9.9)	0.247
Present	36	30 (83.3)	6 (16.7)	
Perceived heath status				
Healthy	179	160 (89.4)	19 (10.6)	0.450
Not healthy	19	16 (84.2)	3 (15.8)	
Daily care for family members				
Present	25	23 (92.0)	2 (8.0)	1.000
Absent	173	153 (88.4)	20 (11.6)	
Parity of their daughter				
Primipara	100	94 (94.0)	6 (6.0)	0.024
Multipara	98	82 (83.7)	16 (16.3)	

Data were analyzed using Fisher's exact test.

† *n* = 197 (no answer from one participant).

Of the 198 grandmothers, 176 (88.9%) supported their daughters and three patterns of support were observed: grandmothers' support at the grandparents' house before childbirth (*n* = 95), grandmothers' support at the grandparents' house after childbirth (*n* = 53), and grandmothers' support at the daughters' house (*n* = 28). Scores of self-esteem and CES-D in each group of support were compared between before and after childbirth (Table 2). Grandmothers who supported their daughters at the grandparents' house before childbirth showed a significant reduction in self esteem score (*P* = 0.009), while there were no significant changes of self-esteem scores of grandmothers in the other three groups. Scores of CES-D did not significantly change before and after childbirth in either subgroup of grandmothers. Furthermore, scores of self-esteem and CES-D before childbirth were compared among support patterns using Tukey's multiple comparison tests. Grandmothers who did not support their daughters showed significantly lower scores of self-esteem than those who provided support at the daughters' house (*P* = 0.021), and also showed significantly lower scores of CES-D than those who provided one of the three patterns of support (at the grandparents' house before childbirth (*P* < 0.001), at the grandparents' house after childbirth (*P* = 0.001), and at the daughters' house (*P* < 0.001), respectively).

**Table 2:** Self-esteem and CES-D scores of grandmothers before and after their daughters' childbirth

Support	N	Self-esteem			CES-D		
		Before	After	*P*-value	Before	After	*P*-value
Total	198	27.2 ± 3.6	27.1 ± 3.8	0.174	8.9 ± 6.7	8.9 ± 6.2	0.883
Grandmothers' support	176	27.4 ± 3.6	27.2 ± 3.8	0.159	8.2 ± 5.6	8.3 ± 5.7	0.690
At the grandparents' house							
Before childbirth	95	27.2 ± 3.3	26.7 ± 3.7	0.009	8.2 ± 5.9	8.7 ± 5.4	0.429
After childbirth	53	27.1 ± 4.3	27.4 ± 4.4	0.558	8.6 ± 5.2	7.9 ± 5.5	0.243
At the daughters' house	28	28.5 ± 3.1	28.3 ± 2.8	0.562	7.0 ± 5.3	7.9 ± 7.1	0.339
No grandmothers' support	22	25.5 ± 3.4	25.4 ± 3.6	0.929	15.0 ± 10.9	13.1 ± 8.3	0.274

Data were analyzed using paired *t*-test. Grandmothers' support at the grandparents' house before childbirth = *Satogaeri bunben*.

Grandmothers were divided into two groups by age: middle-aged and older (Table 3). Middle-aged grandmothers who supported their daughters at the grandparents' house before childbirth showed significantly lower scores after childbirth than before childbirth (*P* = 0.023). In the older group, there were no significant changes in scores of self-esteem before and after childbirth irrespective of the presence or absence of grandmothers' support.

**Table 3:** Self-esteem scores of middle-aged (< 60 years old) and older (≥ 60 years old) grandmothers before and after their daughter's childbirth

Support	Middle-aged				Elderly			
	N	Before	After	*P*-value	N	Before	After	*P*-value
Total	130	27.0 ± 4.0	26.8 ± 4.2	0.398	68	27.6 ± 2.9	27.3 ± 3.1	0.183
Grandmothers' support	114	27.3 ± 3.9	27.1 ± 4.1	0.252	62	27.5 ± 3.0	27.3 ± 3.2	0.401
At the grandparents' house								
Before childbirth	62	27.0 ± 3.7	26.4 ± 3.9	0.023	33	27.7 ± 2.6	27.3 ± 3.4	0.218
After childbirth	39	27.3 ± 4.4	27.5 ± 4.7	0.667	14	26.7 ± 4.0	27.0 ± 3.6	0.612
At the daughters' house	13	29.0 ± 3.4	28.8 ± 3.0	0.730	15	28.1 ± 2.9	27.9 ± 2.6	0.629
No grandmothers' support	16	24.5 ± 3.3	24.9 ± 3.9	0.486	6	28.1 ± 1.7	26.8 ± 2.5	0.102

Data were analyzed using paired *t*-test. Grandmothers' support at the grandparents' house before childbirth = *Satogaeri bunben*.

The Rosenberg self-esteem scale consists of five items of positive attitude and five items of negative attitude. Of 130 middle-aged grandmothers, 114 who supported their daughters and 62 who did so at the grandparents' house before childbirth showed significantly reduced scores in positive attitude after their daughter's childbirth compared with before childbirth, whereas the negative attitude of middle-aged grandmothers was not affected by their daughters' childbirth for any type of grandmothers' support (Table 4).

**Table 4:** Positive and negative attitude in self-esteem scores of middle-aged grandmothers before and after their daughter's childbirth

	N	Positive attitude			Negative attitude		
Before		After	*P*-value	Before	After	*P*-value
Total	130	13.8 ± 1.9	13.5 ± 2.1	0.021	13.1 ± 2.4	13.3 ± 2.4	0.475
Grandmothers' support	114	14.0 ± 1.8	13.7 ± 2.0	0.020	13.3 ± 2.4	13.3 ± 2.4	0.723
At the grandparents' house							
Before childbirth	62	13.8 ± 1.6	13.4 ± 2.0	0.023	13.1 ± 2.5	13.0 ± 2.2	0.470
After childbirth	39	14.0 ± 2.1	13.9 ± 2.2	0.641	13.3 ± 2.6	13.6 ± 2.7	0.274
At the daughters' house	13	15.0 ± 2.0	14.5 ± 1.6	0.190	14.0 ± 1.8	14.3 ± 1.6	0.641
No grandmothers' support	16	12.3 ± 2.0	12.2 ± 1.8	0.817	12.1 ± 1.5	12.6 ± 2.3	0.281

Data were analyzed using paired *t*-test. Grandmothers' support at the grandparents' house before childbirth = *Satogaeri bunben*.

## Discussion

In this study, the provision of grandmothers' perinatal support for their daughters was associated only with parity, indicating that more primiparous women need support from their mother compared with multiparous women. Of the 198 grandmothers, 176(88.9%) provided perinatal support for their daughters in spite of their socioeconomic conditions. In addition, 95 (48.0%) daughters chose to return to their parents' house to give birth. It is suggested that grandmothers play an important role in supporting their daughters, and *Satogaeri bunben* is a common event in modern Japan.

Based on data from Ministry of Health, Labor and Welfare, Japan in 2010, 82.7% of companies adopted mandatory retirement at the age of 60. Grandmothers were thus stratified into two groups: those less than 60 years old (middle aged) and those aged 60 or more (older). Regarding the self-esteem scores of grandmothers before their daughter's childbirth, the middle-aged group scored 27.0 ± 4.0 and the older group 27.6 ± 2.9. In our pilot study using the general population living in the same area as this research, the self-esteem scores of these two age groups were similar to those of the subjects in this study (27.4 ± 3.7 in the middle-aged group and 27.7 ± 2.9 in the older group) (Iseki & Ohashi, 2011). These results indicated that grandmothers who eagerly awaited their daughter's childbirth had the same level of self-esteem as the general population, and the self-esteem of women in these age groups was stable and not affected by their age.

Among the combined, middle-aged, and older groups, self-esteem and depression levels before and after their daughter's childbirth did not change. Shlomo *et al*. (2010) evaluated mental health levels in 102 grandmothers aged 42–62 in Israel and found no significant changes before and after their daughter's childbirth. So, a daughter's childbirth may not influence the grandmother's mental health. However, grandmothers' self-esteem levels, especially of middle-aged grandmothers, who participated in their daughters' *Satogaeri bunben* were significantly reduced after childbirth compared with the time before childbirth. In the middle-aged group, the decreased self-esteem scores were associated with reduction of the five positive items in the tool. Among these five items, the scores of items “On the whole, I am satisfied with myself” and “I take a positive attitude towards myself” significantly decreased (*P* = 0.019 and *P* = 0.027, respectively).

Reitzes and Mutran (1994) reported that self-esteem in middle-aged working women is correlated in part to a person's sense of commitment to adult roles and identity, identifying with their work and family role. In comparison with the older grandmothers, the middle-aged grandmothers showed significantly higher percentages of employment (*P* < 0.001) and daily care for family members (*P* = 0.012), and lower percentages participating in regular hobbies and volunteer activities (*P* = 0.003), suggesting that they might play important roles in home duties as a mother and as a working adult. In the case of *Satogaeri bunben*, a grandmother needs to live together with her daughter and her grandchild/grandchildren for a long time, which automatically increases the burden of housework such as preparing meals and washing clothes for the daughter and grandchildren during the perinatal period. Therefore, the burden on the grandmother increases compared with other perinatal support patterns. As a result, support for grandmothers who take care of their daughters before childbirth is necessary in order to reduce such burdens, especially among middle-aged grandmothers. Other family members and community support may reduce these other burdens. Gerard *et al*. (2006) reported that professional assistance and community services were important in minimizing the negative impact of child-related challenges on grandparents' well-being. A relatively new phenomenon in Japan is the “child-care salon,” offering parental support from older people in the community. A higher frequency of child-care salon participation reportedly reduces child-care-related stress (Kusano *et al.*, 2010).

Self-esteem is an index of mental health well-being and it is considered that supporting others induces a higher level of self-esteem in supporters (Midlarsky, 1991). However, middle-aged grandmothers who participated in their daughters' *Satogaeri bunben* showed significantly reduced levels of self-esteem after childbirth compared with before childbirth. In conclusion, *Satogaeri bunben* is a burden on middle-aged grandmothers and we need to support these grandmothers.

In this study, two questionnaires for grandmothers, self-administered before and after childbirth, were handed to pregnant women at the time of prenatal examinations or mothering classes. To improve the response rate, we asked the post-partum women to urge the grandmothers to respond during hospitalization after childbirth and at their one-month postnatal check by a document. However, the response rate was low (39.5%).

This is the first study to examine the relationship between grandmothers' self-esteem and the perinatal support given to their daughters, so an appropriate sample size could not be determined. In some previous studies investigating the self-esteem of middle-aged/older women, assessed by the Rosenberg scale, the sample sizes ranged between 60 and 300. The sample size for this study was determined based on these reports. Further studies should be conducting using samples with good response rates and adequate sample sizes.

## Conclusion

Grandmothers play an important role in supporting their daughters and grandmothers' support at the grandparents' house before childbirth (*Satogaeri bunben*) is a typical event in modern Japan. Grandmothers, especially middle-aged (less than 60 years old) grandmothers, who supported their daughters at the grandparents' house before childbirth showed significantly lower self-esteem, indicating that *Satogaeri bunben* is a burden for middle-aged grandmothers. We need to support these grandmothers

## Contributions

Study Design: AI, KO.

Data collection and analysis: AI, KO.

Manuscript Writing: AI, KO.
